# Hip and trunk kinematics during reaching on a mobile and stable seat

**DOI:** 10.1371/journal.pone.0289115

**Published:** 2023-07-27

**Authors:** Michelle C. Haas, Bettina B. Sommer, Samuel Karrer, Matthias Jörger, Eveline S. Graf, Martin Huber, Daniel Baumgartner, Christoph M. Bauer

**Affiliations:** 1 School of Health Sciences, Zurich University of Applied Sciences, Winterthur, Switzerland; 2 School of Engineering, Zurich University of Applied Sciences, Winterthur, Switzerland; Kennedy Krieger Institute/Johns Hopkins University School of Medicine, UNITED STATES

## Abstract

Reaching movements are often used to assess selective trunk control in people with neurological conditions. Also, it is known that reaching performance after stroke is increased through training on a mobile seat compared to conventional physical therapy. However, the effect of a mobile seat on joint kinematics has not yet been investigated. This study aimed to quantify differences in the range of motion of the hip and trunk during reaching exercises on a mobile and stable sitting surface. Fifteen healthy participants performed reaching beyond arm’s length on a mobile and a stable seat in four different directions: ipsilateral, anterior, contralateral, and contralateral diagonal. Biomechanical data were collected, including kinematics of the hip and trunk, and surface electromyography of the trunk muscles. The mobile sitting surface led to a higher range of motion in the trunk and the hip in the frontal and sagittal plane, but not in the rotational plane. Differences between reaching directions were found in all joint directions, except that of trunk flexion. Hence, movement patterns of the hip and trunk differ during reaching on different sitting surfaces and in different directions. A larger range of motion in the frontal or sagittal plane while training on the mobile seat provides added neuromuscular stimuli to the trunk muscles (= a higher demand on trunk muscles), which could result in more efficient training and therefore, increased trunk control after stroke. However, this has to be investigated in a future study with people after stroke.

## Introduction

Many activities of daily living, such as putting on socks, tying a shoelace, or reaching for the telephone during office work, are performed in sitting and involve reaching movements [1, 2]. To perform such reaching movements adequately, the ability to selectively control movements of the trunk is required [3]. Reaching also requires coordination of the arm and trunk [4, 5]. When reaching beyond arm’s length interjoint coordination of the shoulder, elbow, wrist, as well as trunk and hip motion is needed [6].

Decreased trunk coordination and limited muscle strength are associated with impairments after stroke [6, 7]. Following a neurological event, such as stroke or spinal cord injury, people often remain impaired in their mobility, especially in the upper extremities, which may be caused by impaired trunk control [8–10]. Even though assessments such as the Fugl-Meyer Scale or the Action Research Arm Test are widely used to assess upper extremity function after stroke, reaching tasks have been shown to correspond with these standard clinical outcome measures [11–13]. According to a review by de los Reyes-Guzman et al. (2014) 77.8% of the reviewed studies assess trunk control of people with neurological conditions is often assessed using functional tasks like reaching movements, reach and grasp movements, or drawing trajectories [12, 14]. In contrast, such functional tasks have not yet been assessed in detail for healthy participants. Instead, assessments have focused on activities of daily living [14]. Thus, when assessing people after stroke using reaching movements, comparable data for healthy participants is lacking.

Diminished selective trunk control in people with neurological conditions could be trained using trunk exercises, such as reaching in sitting [6]. Systematic reviews have shown that, even though muscle activity is increased after sitting balance training on a stable and a mobile sitting surface, mobile sitting might be more efficient to increase trunk control [15, 16]. In addition, training on a mobile seat was found to lead to greater improvement in reaching performance compared to traditional rehabilitation [17]. Using a novel therapy chair, including both mobile and stable seat configurations, trunk control training early after stroke could be made accessible [18]. Since the effect on joint kinematics of the mobile seat has not been investigated yet, a preliminary investigation on healthy participants has been conducted.

The aims of this study were twofold. Firstly, to quantify the differences in the range of motion (ROM) of the hip and trunk between the mobile and stable sitting surface. Secondly, to investigate whether the factors of muscle activity, maximal reaching distance, or reaching direction could explain possible differences in the ROM of the hip and trunk.

## Materials and methods

### Participants

Between September 2020 and March 2021, 15 healthy participants were recruited at the university campus (Winterthur, Switzerland) via mail. Prior to enrolment, screening was made by verbal interview using preselected criteria. The inclusion criteria were over 18 years of age, a body mass index of 18–28 kgm^-2^, and being able to understand verbal and written instructions in German. The exclusion criteria were the presence of acute or chronic musculoskeletal, neurological, or cardiopulmonary diseases, scoliosis, and pregnancy. Participant characteristics are provided in Table 1.

**Table 1:** Participant characteristics.

Characteristic [unit]	Number	Mean	Standard deviation
BMI [kg*m^-2^]	15	23.02	1.57
Age [years]	15	29.87	5.51
Arm length dominant side [cm]	15	75.80	4.41
Sex	5 F / 10 M		
Handedness	13 right, 2 left		

Note. F = Females, M = Males, BMI = Body mass index

### Ethical considerations

The medical Ethics Committee of the Canton of Zurich juristically verified the study (Req-2020-00569), which complied with the tenets of the Declaration of Helsinki. Each participant provided written informed consent prior to the start of the study.

### Apparatus/T-Chair

The mobile seat used in this study was developed to support chronic stroke patients during their sitting balance rehabilitation. Through U-shaped rails, it has the ability to move in the sagittal and frontal plane, or a combination thereof, thus allowing a 3D range of movement [18]. The seat can be used in a locked position, referred to as the stable seat condition, or in an unlocked position, referred to as the mobile seat condition.

### Reaching tasks

**Fig 1:**
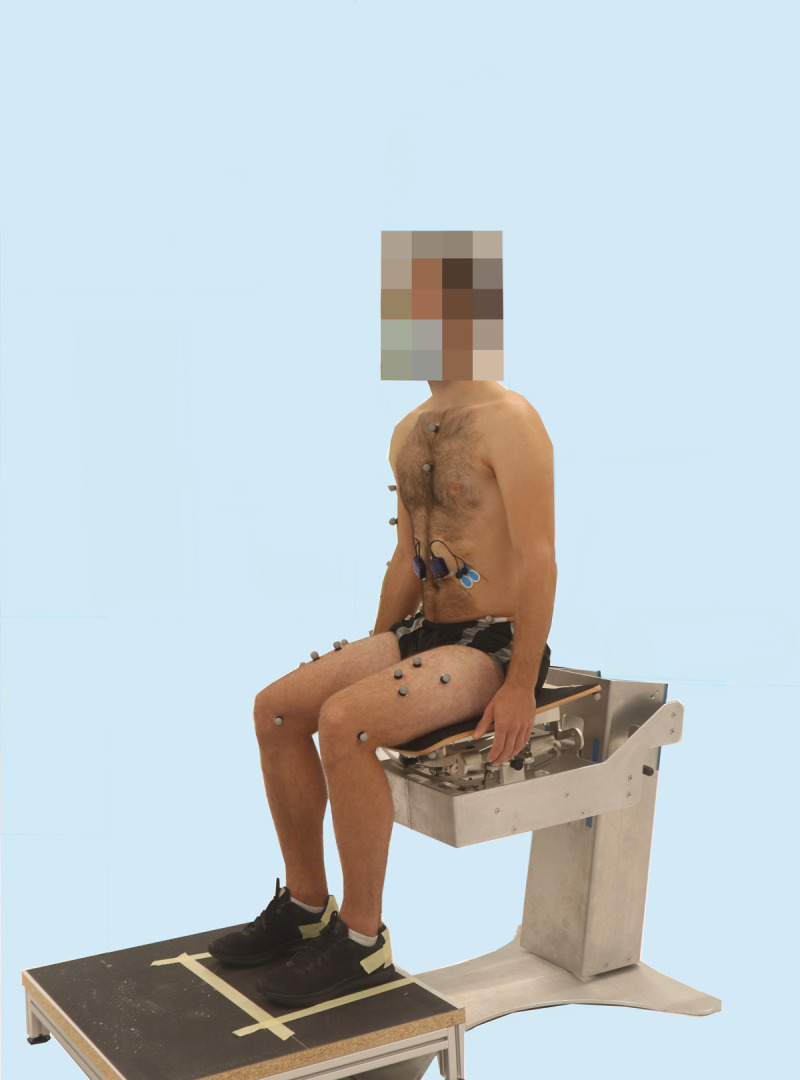
Starting position. Participant sitting in the starting position on the stable seat.

**Fig 2:**
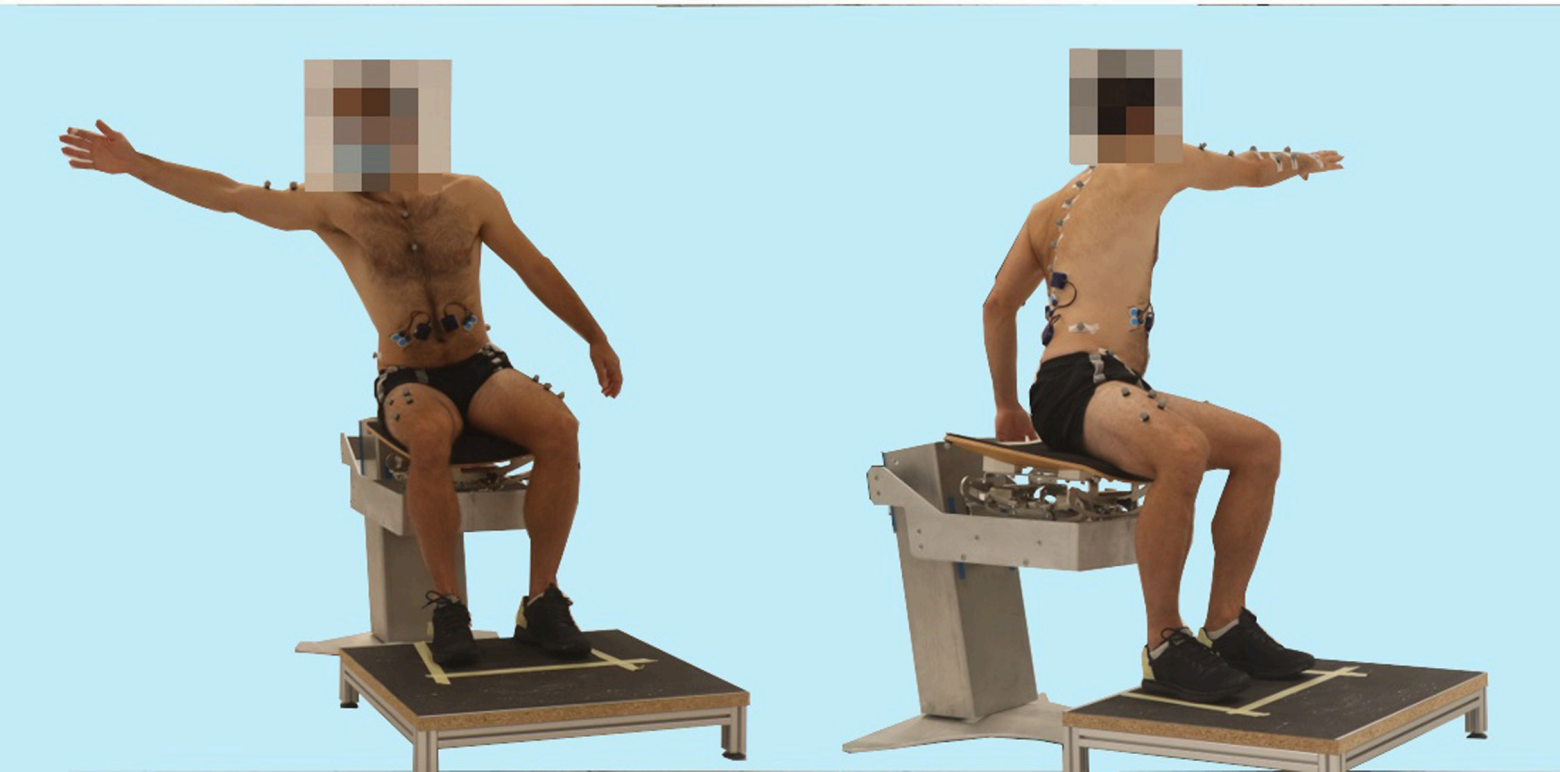
Reaching tasks. Ipsilateral reaching (left) and contralateral reaching (right).

**Fig 3:**
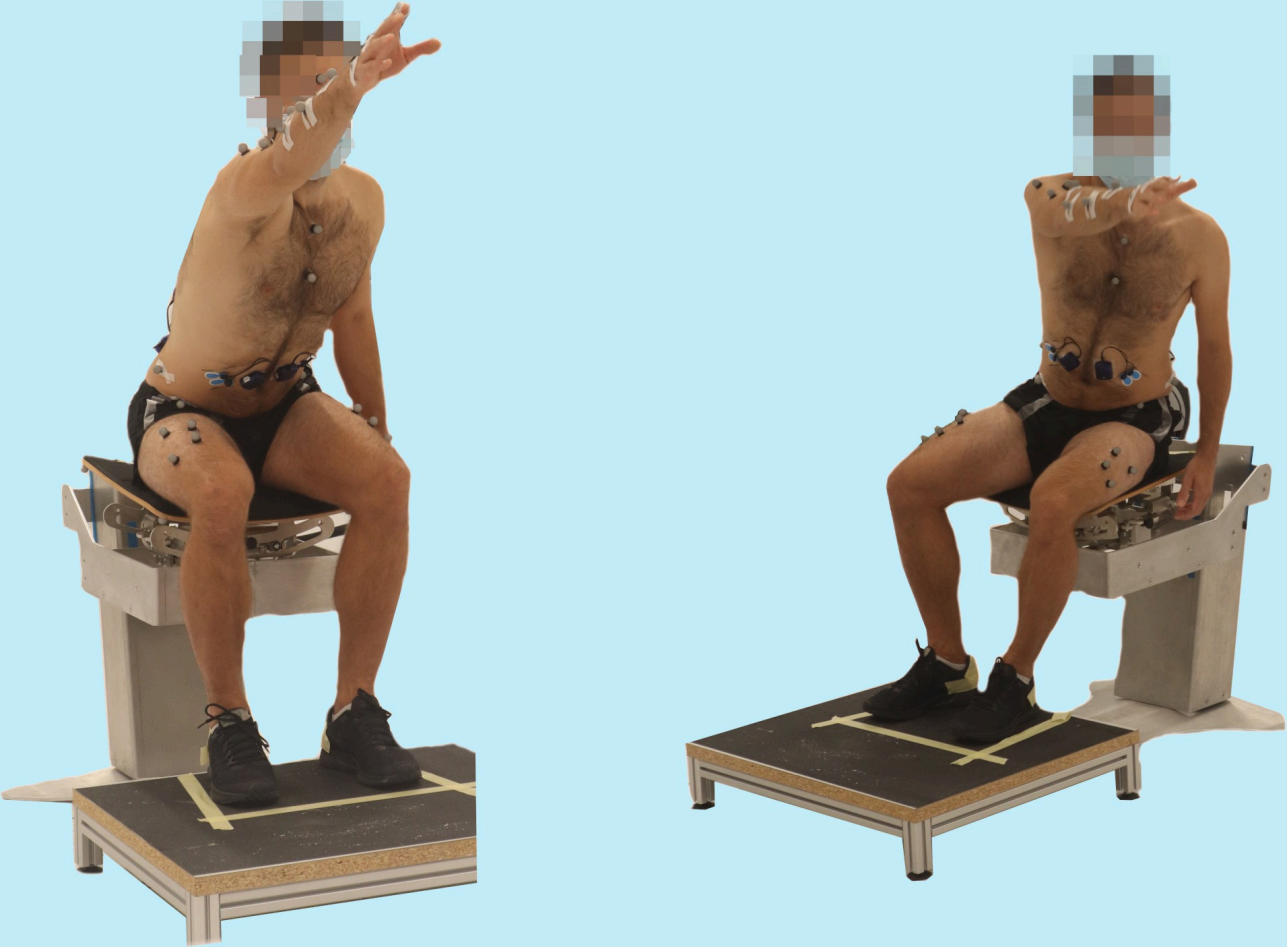
Reaching tasks. Anterior reaching (left) and contralateral diagonal reaching (right).

The starting position for each reaching task was sitting upright, with the hip and knees at a 90° angle, feet hip-width apart and placed on a footstool (Fig 1). For contralateral movements, the dominant arm was placed on the contralateral leg with the non-dominant arm hanging loosely at the side of the body. For ipsilateral and anterior movements, both arms hung loosely at the side of the body with the hands next to the hips. Reaching was performed beyond arm’s length using the dominant arm. To assure reaching distance beyond arm’s length, participants were required to reach to a pylon in a test trial, which was then placed further away for the trials. For each task, participants were instructed to reach to the pylon in the direction of an arrow on the floor. Reaching tasks were performed with five repetitions in four different directions. During contralateral reaching, the dominant arm reached to the non-dominant side in the frontal plane (Fig 2). For contralateral diagonal reaching, the participant was instructed to reach diagonally to the contralateral side. In anterior reaching, the participant reached anteriorly in the sagittal plane (Fig 3). During ipsilateral reaching, the dominant arm reached to the dominant side in the frontal plane (Fig 2).

Time standardization of the task was performed as following. Starting position was considered as 0% of task time, while returning to the starting position after the reach was defined as 100% of task time.

### Data collection and analysis

Data collection took place in the movement laboratory of Zurich University of Applied Sciences. Arm length was measured from the acromion to the pointing finger with the arms hanging loosely next to the body. Reflective markers were placed on the dominant side of the participant’s upper arm and pointing finger, as well as on the thigh, pelvis, and trunk. The markers on the thigh, pelvis, and upper arm were placed according to the cluster marker model of List et al. (2013). The marker model of Rast et al. (2016) [19] was used for the trunk. In total 21 markers were attached to a participant’s skin. For angle calculations of the trunk (movement between thorax and pelvis) and hip (movement between thigh and pelvis) a segmental approach was used, resulting in three output angles: flexion, lateral flexion (trunk) or adduction (hip), and rotation. Positive angular values were assigned to flexion, lateral bending to the right side/adduction, and counterclockwise rotation. The position of the shoulder joint at the starting position, which was calculated through a functional calibration task similar to the hip joint [20], was the reference point for calculation of the maximal reaching distance (maxreach). Reaching distance was calculated as the vector from the reference point to the pointing finger for each frame and the maximal resulting vector was extracted as maxreach. Maxreach was normalized to each participant’s arm length with the participant’s arm length representing 100%.

After shaving and cleaning of the skin, bipolar electrodes (Blue Sensor, Ambu, Denmark, Type P-00, interelectrode distance: 20.148 mm) were placed bilaterally at the M. Multifidi (MF), M. Erector Spinae (ES), and M. Obliquus Externus (OE). Electrodes were placed according to the recommendations of the surface electromyography (sEMG) of the non-invasive assessment of muscle project and Ng and colleagues [21, 22]. The wireless myon sEMG system (myon AG, Baar Switzerland; Type 142 RFTD-A01, D02-RFTD) was used to record the sEMG signal at a sampling rate of 1200Hz and 12-bit resolution. All data (Kinematics & sEMG) were collected using an infrared, camera-based motion capture system (Vicon, Oxford. UK, Version 2.11). Data were post-processed with MATLAB (MathWorks Inc. Natick, MA, USA, Version R2019a). A 4^th^ order Butterworth filter with a cut-off frequency of 7 Hz was applied to the marker data. Post-processing of sEMG-data was performed as in a previous study using the mean of the five repetitions as outcome [23, 24]. Maximal root mean square (RMS) sEMG values were put in relation to maximal RMS sEMG values during static sitting using the following formula.


$$\;Maximal\;RMS\;sEMG\;(\text{\%}STAT)\;=\;\frac{\;maximal\;RMS\;sEMG\;(\;trial\;in\;mV\;)\;*\;100\text{\%}}{\;maximal\;RMS\;sEMG\;(\;static\;sitting\;in\;mV)}$$

### Statistical analysis

The mean ROM of the five repetitions of each angle was used as the outcome variable for statistical analysis. In hip kinematics, the dominant side was used for statistical analysis since it can be assumed that the movement of one side of the hip depends on the other side’s movement. Included co-variates were maxreach as well as maximal RMS sEMG in percentage of the static sitting trial. Linear-mixed models were used for all outcome variables with a significance level of p < .05. Firstly, all covariates (MF, ES, OE, maxreach) were included in the model. Then, through backwards optimization, the model with the best fit (evaluated with the Akaike Information Criterion) for all three directions of the joint angle was found and the same models were applied to all joint directions. An overview of the tested models can be found in the S2 File. Distribution of the residuals was visually analyzed in order to ensure model fit. Due to residual analysis the outcome variable was logarithmically transformed. The following final models, with Y representing the outcome of interest, were used:

$$\begin{array}{l} -\;\text{Hip}:\;Y\;=\;Condition\;+\;Exercise\;+\;Maxreach\;+\;OE_{dominant}\;+\\ \text{(1|}Condition:\;Subject\;ID)\;+\;(1|Exercise\;:\;Subject\;ID)\;+\;(1|Subject\;ID) \end{array}$$

$$\begin{array}{l} -\;\text{Trunk}:\;Y\;=\;Condition\;+\;Exercise\;+\;Maxreach\;+\;ES_{non-dominant}\;+\;OE_{non-dominant}\;+\\ \text{(1|}Condition:\;Subject\;ID)\;\text{+}\;(1|Exercise:\;Subject\;ID\text{)} \end{array}$$

After application of the statistical model, marginal means were estimated (also called least-squares means) to predict the ROM of future data using the function emmeans. R Version 4.10 [25], including the packages lme4 [26], lmerTest [27], emmeans [28], and ggplot2 [29], was used to calculate all statistical analyses.

## Results

Descriptive values as well as joint angle illustration plots for the ROM of the hip and trunk in all four reaching directions, can be found in the S1 File.

### Hip

Differences in the ROM of hip adduction between the mobile and the stable seat condition were small and not significant, although the ROM tended to be slightly higher on the mobile seat. While in the anterior and ipsilateral directions the predicted ROM was smaller than 6°, in the contralateral and the contralateral diagonal directions the predicted ROM was between 10° and 15° (Table 2). The predicted ROM for hip adduction showed a significant main effect for the reaching direction, F (3, 52.36) = 56.51, p < .001 and for the dominant side of OE F (1, 89.94) = 7.22, p = .009 (*R*^2^ = .82).

**Table 2:** Predicted hip range of motion for each movement and reaching direction on the mobile and stable seats.

	Adduction						Flexion						External rotation					
Reaching direction	Mobile seat			Stable seat			Mobile seat			Stable seat			Mobile seat			Stable seat		
	Predicted ROM [°]	95% CL		Predicted ROM [°]	95% CL		Predicted ROM [°]	95% CL		Predicted ROM [°]	95% CL		Predicted ROM [°]	95% CL		Predicted ROM [°]	95% CL	
		Lower	Upper		Lower	Upper		Lower	Upper		Lower	Upper		Lower	Upper		Lower	Upper
Contralateral	15.13	11.64	19.66	13.73	10.70	17.63	10.18	7.53	13.77	6.94	5.26	9.16	7.84	6.22	9.90	8.49	6.85	10.52
Contralateral- diagonal	12.31	9.57	15.83	11.17	8.54	14.61	17.60	13.28	23.33	11.99	8.75	16.42	6.38	5.13	7.93	6.90	5.42	8.80
Anterior	4.26	3.14	5.77	3.86	2.74	5.45	28.59	19.52	41.86	19.48	12.42	30.56	5.70	4.25	7.64	6.17	4.37	8.71
Ipsilateral	5.80	4.05	8.31	5.27	3.84	7.23	15.30	9.50	24.65	10.43	6.95	15.63	12.94	8.98	18.64	14.00	10.26	19.11

ROM = Range of motion, CL = Confidence Level

**Fig 4:**
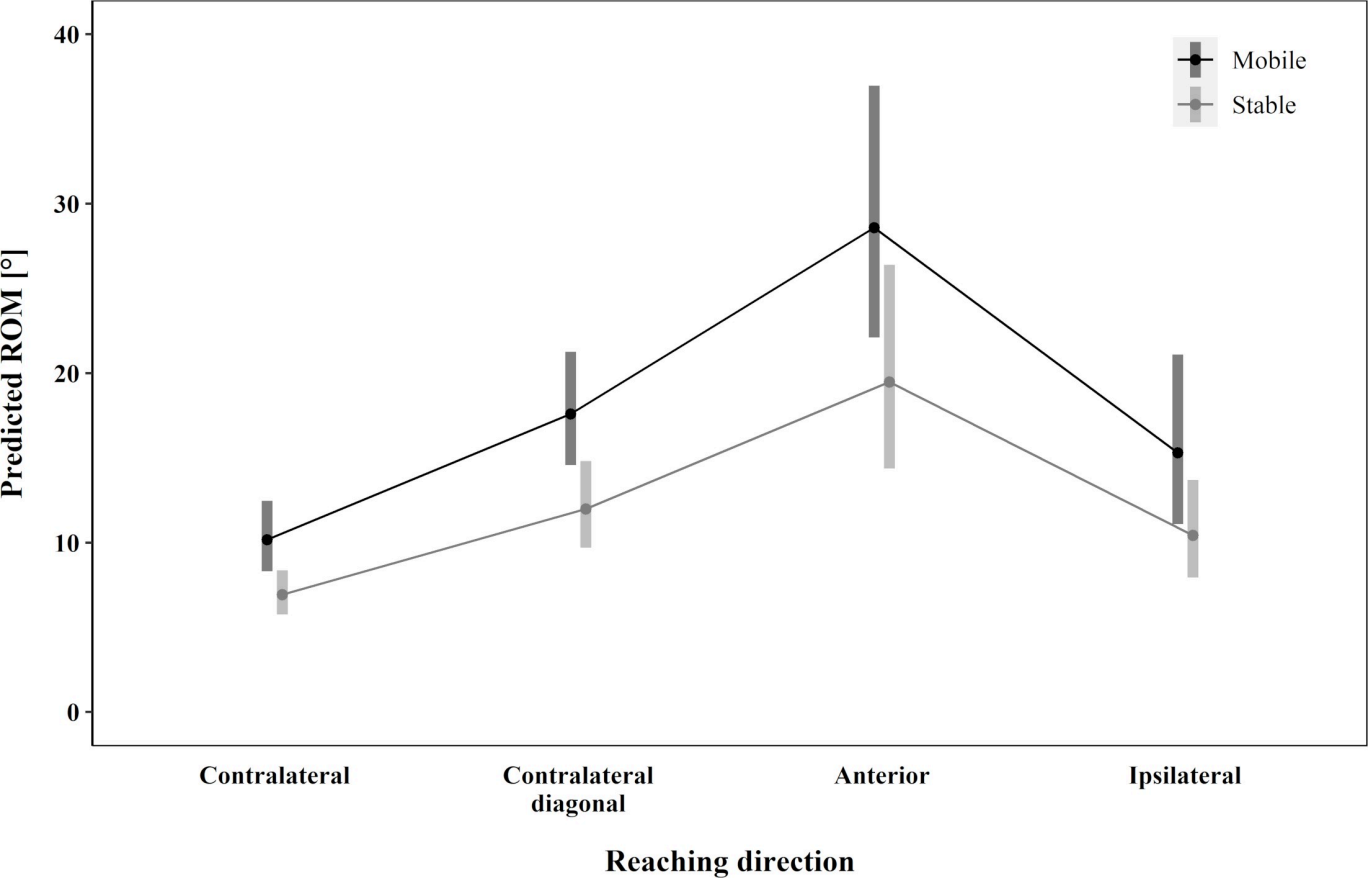
Hip flexion. The predicted range of motion for hip flexion.

The predicted ROM for hip flexion was higher during mobile sitting in all reaching directions. Anterior reaching showed the most prominent differences between the mobile and stable seats. In addition, anterior reaching showed the highest predicted ROM (Fig 4). Ipsilateral and contralateral diagonal reaching showed a similar predictive ROM for the mobile and stable seats. Although during contralateral reaching the difference between the two seat conditions was similar to that of ipsilateral and contralateral diagonal reaching, the predicted ROM was the lowest (Table 2). In hip flexion significant main effects for seat condition F (1, 65.21) = 26.25, p < .001, reaching direction F (3, 50.90) = 21.42, p < .001, and maxreach F (1, 92.96) = 19.13, p < .001 were found (*R*^2^ = .80).

A tendency towards a higher predicted ROM of external hip rotation was found in the stable sitting condition, but the difference was insufficient to result in a significant main effect. Significant main effects were found for reaching direction F (3, 50.51) = 9.57, p < .001 and maxreach F (1, 89.58) = 15.13, p < 0.001 (*R*^2^ = .54). On the one hand, differences between the seat conditions in all reaching directions were small, but on the other hand, the predicted ROM was higher in the ipsilateral reaching direction compared to the other directions (Table 2).

### Trunk

As with hip adduction, predicted differences between the mobile and stable seats were small for trunk lateral flexion and not significant. Again, the tendency was towards slightly higher values on the mobile seat. Significant main effects were found for the reaching direction F (3, 53.31) = 43.21, p < .001 and maxreach F (1, 84.26) = 7.17, p = .009 (*R*^2^ = .85). The highest predictive ROM can be seen during contralateral reaching, followed closely by contralateral diagonal reaching. A few degrees less were predicted for ipsilateral and for anterior reaching (Table 3).

**Table 3:** Predicted trunk range of motion for each movement and reaching direction on the mobile and stable seats.

	Lateral flexion						Flexion						Rotation					
Reaching direction	Mobile seat			Stable seat			Mobile seat			Stable seat			Mobile seat			Stable seat		
	Predicted ROM [°]	95% CL		Predicted ROM [°]	95% CL		Predicted ROM [°]	95% CL		Predicted ROM [°]	95% CL		Predicted ROM [°]	95% CL		Predicted ROM [°]	95% CL	
		Lower	Upper		Lower	Upper		Lower	Upper		Lower	Upper		Lower	Upper		Lower	Upper
Contralateral	21.59	15.89	29.35	19.85	14.89	24.47	14.06	9.59	20.61	10.66	7.38	15.41	36.01	27.36	47.39	39.03	29.99	50.78
Contralateral- diagonal	18.96	14.18	25.37	17.44	12.74	23.86	11.37	7.85	16.47	8.62	5.85	12.70	24.77	19.00	32.30	26.85	20.31	35.50
Anterior	4.97	3.40	7.27	4.57	2.95	7.10	14.02	9.01	21.82	10.63	6.48	17.46	6.78	4.92	9.35	7.35	5.12	10.56
Ipsilateral	15.29	9.77	23.94	14.06	9.56	20.69	13.63	8.23	22.56	10.33	6.60	16.18	9.86	6.83	14.25	10.69	7.72	14.81

ROM = Range of motion CL = Confidence Level

Differences in trunk flexion between the seat conditions were around 3–5° for all reaching directions, with a greater predicted ROM during mobile sitting. The main effect of trunk flexion was significant for seat condition F (1, 16.61) = 12.67, p = .002 and maxreach F (1, 94.90) = 9.61, p = .003 (*R*^2^ = .77). Reaching direction showed no significant main effect. A similar ROM was predicted for contralateral, anterior, and ipsilateral directions. The diagonal contralateral direction showed a smaller predicted ROM (Table 3).

**Fig 5:**
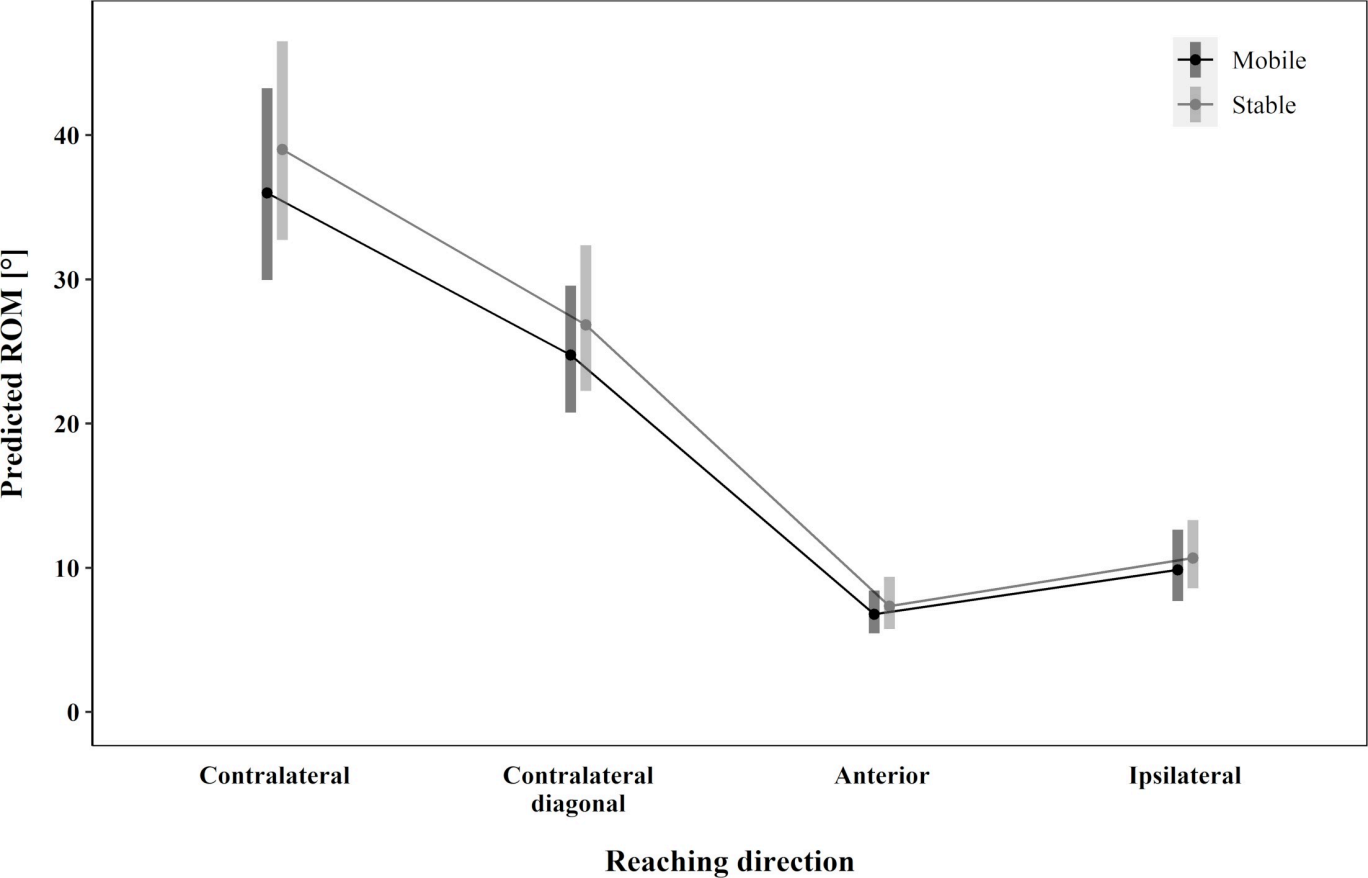
Trunk rotation. The predicted range of motion for trunk rotation.

For all reaching directions, the stable seat tended towards higher values for trunk rotation compared to the mobile seat (Fig 5). However, the differences were small, and no significant main effect was found. Reaching direction F (3, 56.52) = 112.50, p < .001, maxreach F (1, 83.60) = 10.04, p = .002, OE non-dominant side F (1, 73.96) = 4.68, p = .03, and ES non-dominant side F (1, 93.62) = 4.79, p = .03 showed significant main effects for trunk rotation (*R*^2^ = .94). Overall, the greatest ROM was predicted for contralateral reaching, followed by contralateral diagonal, ipsilateral and anterior reaching directions (Table 3).

Maxreach was higher on the stable seat for all reaching directions, but the difference was very small for anterior reaching (Table 4).

**Table 4:** Maxreach (Mean ± standard deviation [percentage of arm length]).

Reaching direction	Mobile Seat	Stable Seat
Contralateral	131.82 ± 10.03	144.71 ± 6.07
Contralateral diagonal	148.45 ± 8.24	152.17 ± 10.66
Anterior	163.19 ± 9.88	163.30 ± 11.27
Ipsilateral	116.05 ± 4.43	125.15 ± 5.84

## Discussion

Differences between mobile and stable sitting surfaces in the ROM of the hip and trunk were quantified. A significant effect of the seat condition was found for hip and trunk flexion. Also, it was investigated whether the factors of muscle activity, maxreach, or reaching direction could explain differences in ROM. In trunk rotation, muscle activity of the non-dominant side of OE and ES affected the ROM. Additionally, the dominant side of OE affected hip adduction. Reaching direction influenced the ROM for all joints and directions, except for trunk flexion. Similar results were found for maxreach, where effects were significant for all joints and directions, apart from hip adduction. Therefore, depending on the joint and its movement direction, a combination of muscle activity, maxreach, and reaching direction can explain differences in the ROM.

Predicted ROM tended to be higher on the mobile seat for hip flexion, hip adduction, trunk flexion, and trunk lateral flexion. However, only flexion of the hip and trunk showed a statistically significant effect. The mobile seat allows more degrees of freedom, which possibly explains the decreased ROM on the stable seat [18]. The ROM tended to be higher on the stable seat for hip external rotation and trunk rotation, which contradicts this assumption. Possibly, a greater amount of stabilization from the muscles is needed on the mobile seat and therefore, the hip and trunk rotate less due to muscular effort limits. To maintain stability on the mobile seat, the hip would have to counteract the rotation of the trunk and vice versa or rotation being the most unstable movement direction would explain a higher ROM on the stable seat. Due to added training stimuli for the trunk muscles through a higher ROM, the mobile seat could be more efficient to increase trunk control after stroke [30].

Maxreach was higher on the stable seat, independent of exercise, which could be explained by greater stability of the body on the stable seat. Steadiness of the body is lower with higher muscle tension and, therefore, muscle stiffness on the mobile seat could lead to lower maxreach values [31]. With increasing maxreach, foot support and muscle activation in the legs increase [32]. On the mobile seat, leg muscles assist in stabilization from the beginning. This could mean that the additional capability of support from the legs is limited because their strength limit is reached earlier. Consequently, maxreach is smaller which implies the suitability of reaching exercises on the mobile seat after stroke. With lower maxreach, the center of pressure remains over the base of support (BoS) longer, leading to higher stability. BoS is defined as the foot placement area plus the contact area of the buttocks to the sitting surface. Since reaching improves following mobile sitting training, it is possible that maxreach would increase over time [17].

Trunk muscles can only explain the differences in ROM for hip adduction and trunk rotation. Since reaching was performed with the dominant arm and therefore, in most reaching exercises rotation was executed to the non-dominant side, the significance of the non-dominant side in trunk rotation is not surprising. In a previous study, differences in sEMG were found for trunk control exercises in a healthy participants as well as in people after stroke [23]. Because all these effects were very small and high confidence levels showed high variability in muscle activity, the interpretation of these effects will not be further elaborated.

Reaching direction showed a main effect in all planes and joints, except for trunk flexion, which may be due to different limits of stability. During trunk flexion, the ROM stayed at the same level under all seat conditions and reaching exercises. Likely, the trunk needs to be flexed to a certain extent to correctly perform the reaching movement beyond arm’s length. This “minimal” flexion seems to be consistent across seat conditions and reaching directions. Lateral reaching assesses medial-lateral components of postural control, anterior reaching assesses anterior-posterior components, and diagonal reaching assesses a combination thereof [33]. This suggests different demands on postural control that can be trained during rehabilitation after stroke.

Differences regarding reaching direction could also be explained by the center of mass shifting away from the support base earlier in lateral reaching (in this case contralateral and ipsilateral) than in anterior reaching [34]. Hof et al. (2005) [35] defined dynamic stability as the center of mass relative to the BoS. The BoS is higher on the mobile seat because the contact area to the sitting surface stays at the same level independent of the movement on the seat. Therefore, higher dynamic stability is expected on the mobile seat. Due to foot placement, the highest BoS is present in anterior reaching and the lowest BoS in ipsilateral reaching. This means that dynamic stability is highest in the anterior and lowest in ipsilateral reaching. ROM and maxreach are expected to be higher with greater dynamic stability. This assumption was confirmed for maxreach, but for ROM only in hip flexion. In hip adduction and all planes of the trunk, ROM was highest during contralateral reaching and in hip rotation during ipsilateral reaching. This implies that anterior reaching, due to its high dynamic stability, could be performed earlier than all other reaching directions during trunk rehabilitation after stroke.

Combining the predicted ROM of the trunk and hip results in distinct movement patterns for each reaching direction. The ROM of the hip during ipsilateral reaching differs between the mobile and stable seat in the lateral and rotational components, in the anterior component it is the trunk. Therefore, the mobile seat is compensated by either the hip or the trunk. Rotation of the trunk and hip, as well as hip adduction and trunk lateral flexion, were probably smallest during anterior reaching because the movement is unidirectional. While flexion of the trunk during anterior reaching is similar to other directions, flexion of the hip is high. During anterior reaching the BoS through the feet is high, which leads to higher muscle activation of the legs, lower muscle activation of the trunk, but higher stability [31, 32, 36]. Additionally, the center of mass shifts away from the BoS near maxreach [34]. Contralateral reaching is performed mainly in the frontal plane resulting in low flexion of the trunk and hip. While the movement of the trunk and hip in the lateral direction and hip rotation are small, the ROM of trunk rotation was highest in this task. In the lateral direction, the target is placed far away from the pointing finger and much rotation from the trunk is needed to reach the target. To avoid falling, the hip stabilizes that movement and is kept at a low ROM. For contralateral diagonal reaching, movement in all planes was expected, but not confirmed by the data. ROM in the frontal plane and hip rotation were small. However, a large ROM for trunk rotation was found. Trunk flexion was similar to contralateral reaching, but hip flexion was somewhat higher than in contralateral reaching. Higher hip flexion angles could be explained by the more anterior placement of the target, leading to a larger involvement of the sagittal plane.

Possible limitations of this study were the uncontrolled target height and speed of the reaching movement. Since, however, reaching was performed to a pylon with a standardized height and the task time was normalized, it can be assumed that this did not affect the ROM of the hip and trunk. Due to the Covid-19 pandemic only a small, young and physically fit sample size could be recruited. This does not represent the general world population. It is known from literature that the average distance reached, as well as the lateral pelvic tilt range, decreases with increasing age [37]. Due to the young study population, it can be assumed that age-related conditions, such as soft tissue shortening or reduced muscle strength, did not influence maxreach. Another limitation is that there were more male than female participants, and potential gender effects have not been considered. Males and females have different fat distributions, which could affect muscle activity measurements. Additionally, the handedness of participants was unbalanced. Only two out of the fifteen participants had a dominant left hand. The number of left-handed people was so small that it is not expected to have made a difference.

## Conclusion

A mobile sitting surface leads to a higher ROM of the trunk and the hip in the frontal and sagittal planes, but not in the rotational plane. When a larger range of motion in the frontal or sagittal plane is desired, the mobile seat would be a good training tool for trunk rehabilitation since there would be additional training stimuli. However, this has to be investigated in a future study of kinematics on the mobile and stable seat in people after stroke.

## Supporting information

S1 Fig Comparison of contralateral reaching on the stable and mobile seat. (TIF)

S2 Fig Comparison of contralateral-diagonal reaching on the stable and mobile seat. (TIF)

S3 Fig Comparison of anterior reaching on the stable and mobile seat. (TIF)

S4 Fig Comparison of ipsilateral reaching on the stable and mobile seat. (TIF)

S1 File Supplementary data. (DOCX)

S2 File Supplementary data and statistical analysis. (DOCX)
