# Supplementary figures and images for: Hip and trunk kinematics during reaching on a mobile and stable seat
Source: PLoS One. 2023 Jul 27;18(7):e0289115. doi: 10.1371/journal.pone.0289115 (PMC10374116; doi:10.1371/journal.pone.0289115)

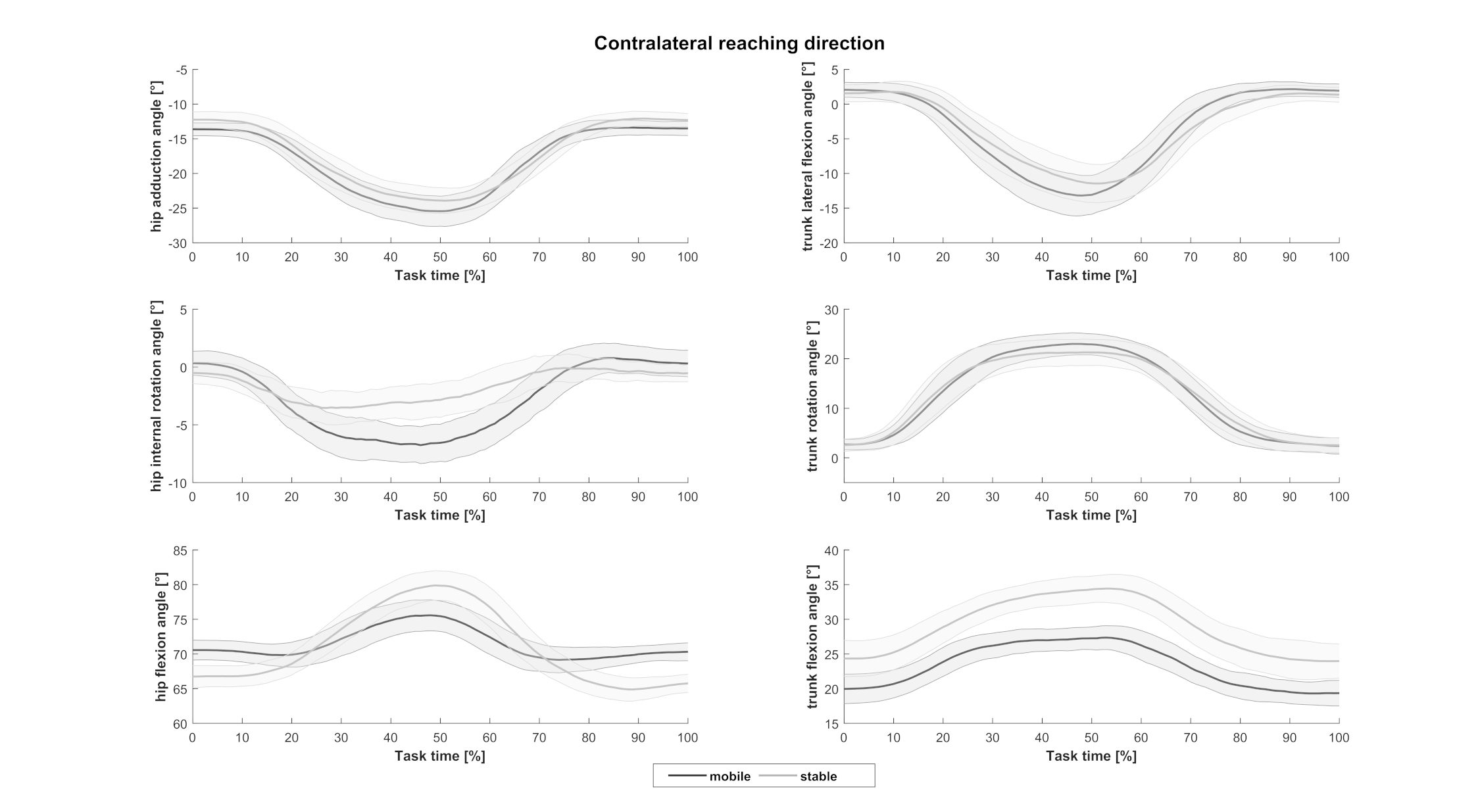

Supplement: S1 Fig — (TIF) [file pone.0289115.s001.tif]

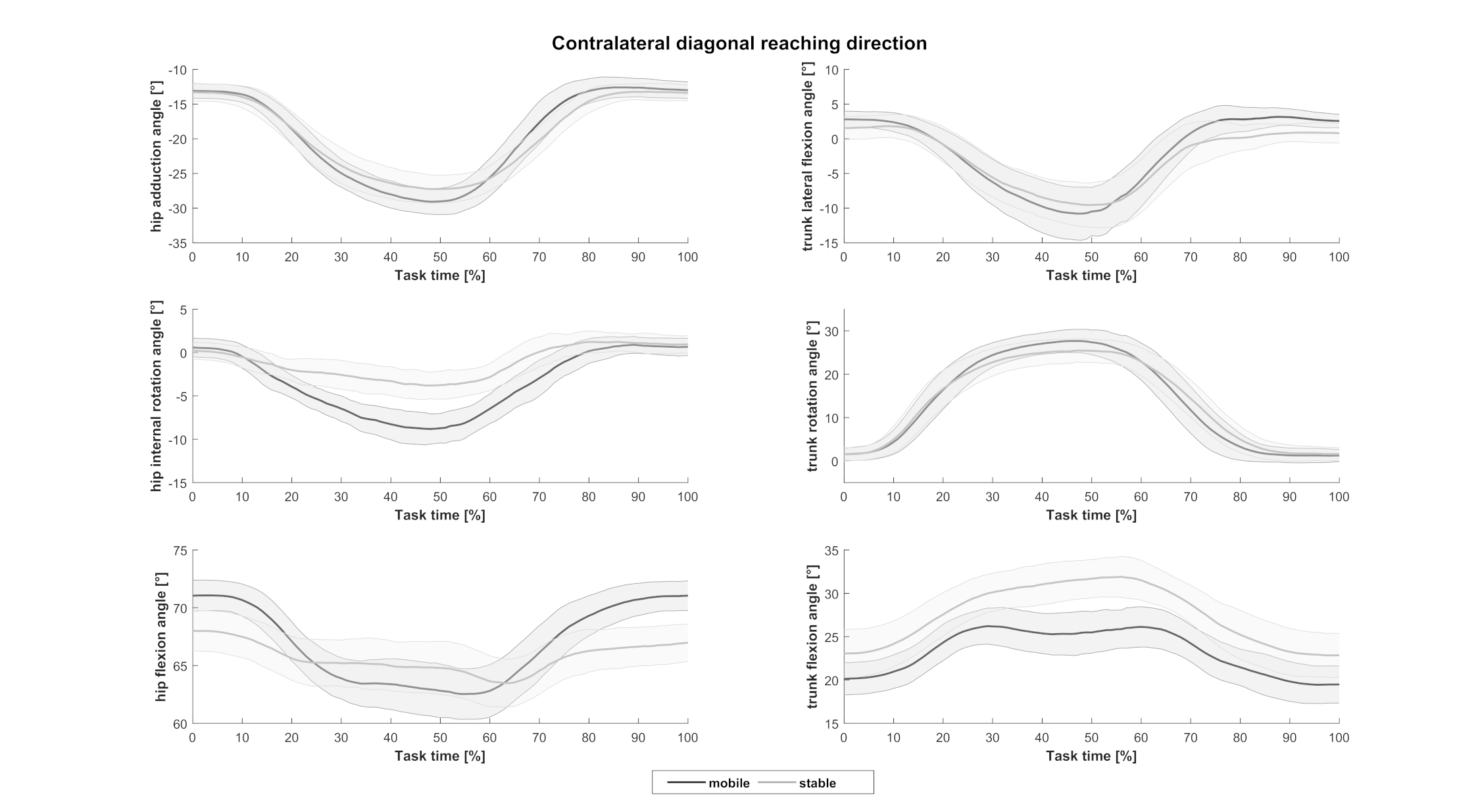

Supplement: S2 Fig — (TIF) [file pone.0289115.s002.tif]

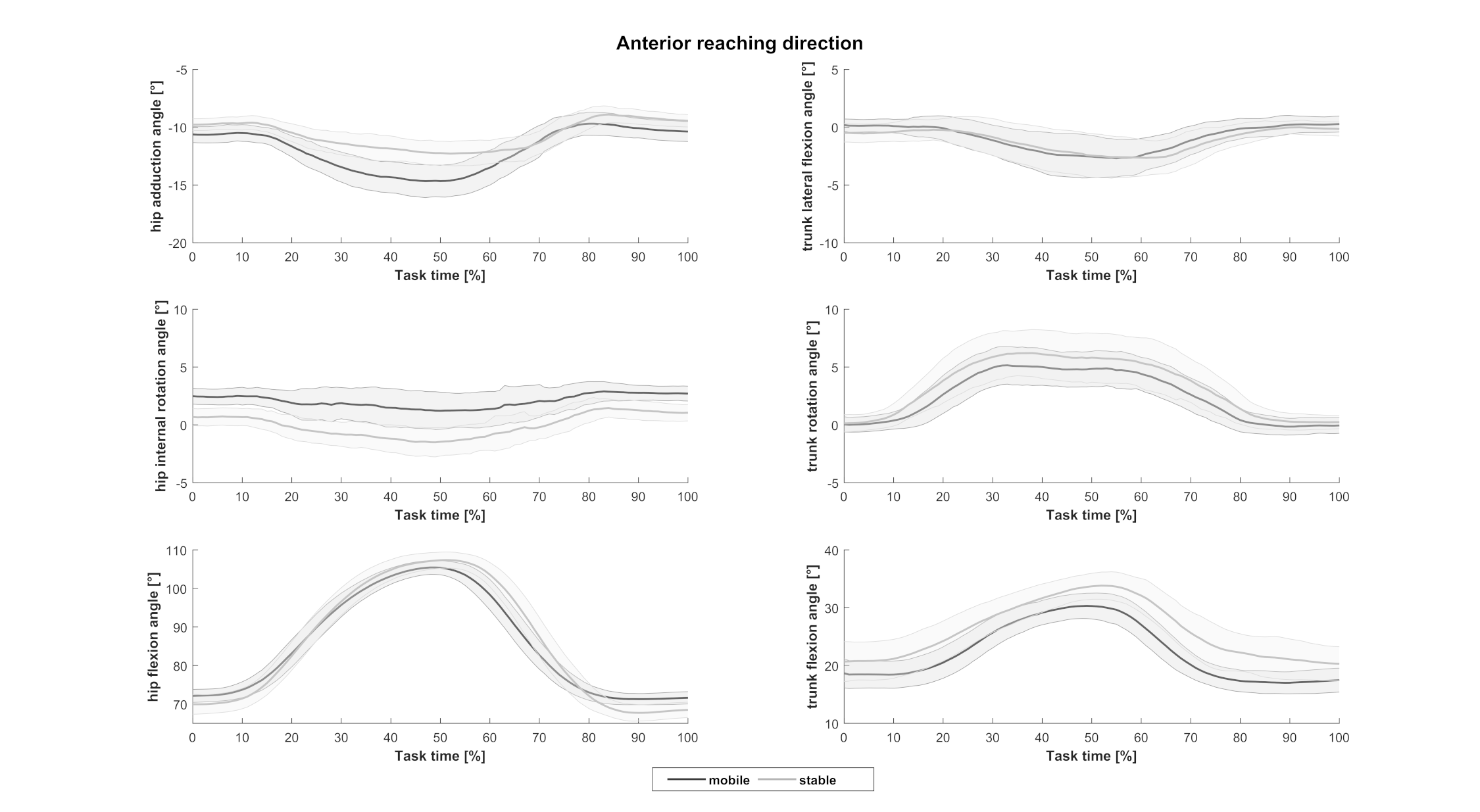

Supplement: S3 Fig — (TIF) [file pone.0289115.s003.tif]

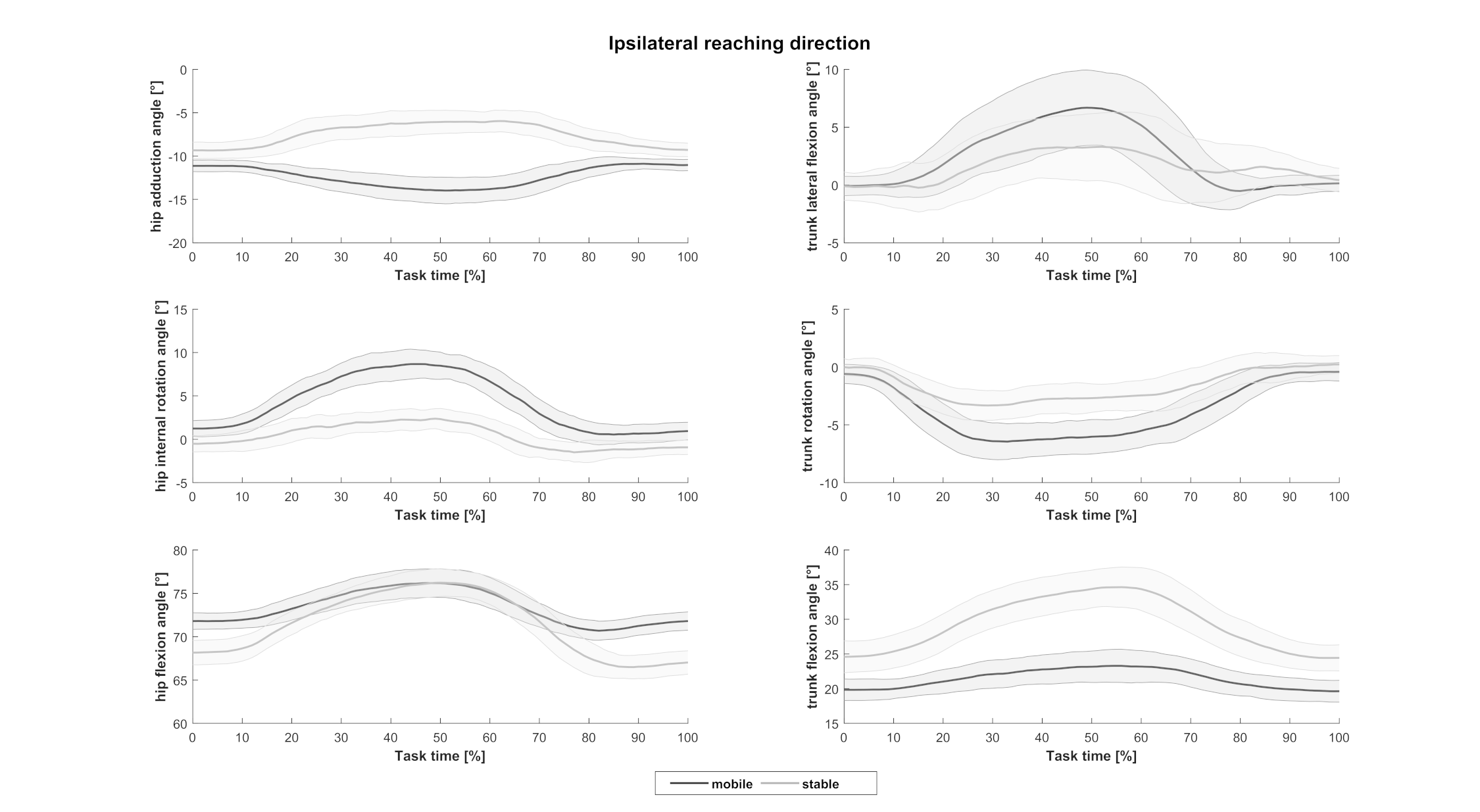

Supplement: S4 Fig — (TIF) [file pone.0289115.s004.tif]
