# Supplementary material for: Hip and trunk kinematics during reaching on a mobile and stable seat
Source: PLoS One. 2023 Jul 27;18(7):e0289115. doi: 10.1371/journal.pone.0289115 (PMC10374116; doi:10.1371/journal.pone.0289115)
Supplement: S1 File — (DOCX) [file pone.0289115.s005.docx]

# Supplementary Material

Descriptive values for each joint (real means)

Hip adduction

| Seat | Reaching direction | N | Mean angle [°] | SD | Minimal angle [°] | Maximal angle [°] |
| --- | --- | --- | --- | --- | --- | --- |
| Mobile | Contralateral | 15 | 15.76 | 7.17 | 6.07 | 29.05 |
|  | Contralateral Diagonal | 15 | 14.51 | 7.36 | 5.38 | 32.4 |
|  | Anterior | 15 | 4.48 | 1.71 | 2.08 | 8.85 |
|  | Ipsilateral | 15 | 6.07 | 3.60 | 2.41 | 15.91 |
| Stable | Contralateral | 15 | 16.42 | 8.01 | 6.91 | 33.31 |
|  | Contralateral Diagonal | 15 | 12.48 | 7.38 | 4.03 | 30.22 |
|  | Anterior | 15 | 5.34 | 4.43 | 2.22 | 12.21 |
|  | Ipsilateral | 15 | 4.66 | 2.00 | 1.59 | 9.09 |

Hip flexion

| Seat | Reaching direction | N | Mean angle [°] | SD | Minimal angle [°] | Maximal angle [°] |
| --- | --- | --- | --- | --- | --- | --- |
| Mobile | Contralateral | 15 | 9.98 | 5.15 | 4.09 | 21.98 |
|  | Contralateral Diagonal | 15 | 22.36 | 6.94 | 12.81 | 38.00 |
|  | Anterior | 15 | 39.62 | 7.18 | 24.64 | 55.40 |
|  | Ipsilateral | 15 | 9.01 | 4.00 | 3.61 | 15.73 |
| Stable | Contralateral | 15 | 6.98 | 2.99 | 3.21 | 15.68 |
|  | Contralateral Diagonal | 15 | 15.01 | 6.82 | 3.88 | 29.96 |
|  | Anterior | 15 | 33.37 | 6.07 | 25.12 | 44.85 |
|  | Ipsilateral | 15 | 9.28 | 4.45 | 1.50 | 17.97 |

Hip external rotation

| Seat | Reaching direction | N | Mean angle [°] | SD | Minimal angle [°] | Maximal angle [°] |
| --- | --- | --- | --- | --- | --- | --- |
| Mobile | Contralateral | 15 | 7.49 | 2.84 | 3.87 | 13.72 |
|  | Contralateral Diagonal | 15 | 7.27 | 2.05 | 3.43 | 11.86 |
|  | Anterior | 15 | 7.53 | 2.75 | 3.74 | 13.59 |
|  | Ipsilateral | 15 | 8.88 | 3.11 | 4.77 | 16.70 |
| Stable | Contralateral | 15 | 8.23 | 2.69 | 3.73 | 12.66 |
|  | Contralateral Diagonal | 15 | 8.06 | 3.48 | 4.14 | 16.07 |
|  | Anterior | 15 | 8.68 | 3.15 | 4.94 | 14.04 |
|  | Ipsilateral | 15 | 12.64 | 2.62 | 6.83 | 16.62 |

Trunk flexion extension

| Seat | Reaching direction | N | Mean angle [°] | SD | Minimal angle [°] | Maximal angle [°] |
| --- | --- | --- | --- | --- | --- | --- |
| Mobile | Contralateral | 15 | 13.15 | 7.03 | 5.51 | 32.69 |
|  | Contralateral Diagonal | 15 | 13.66 | 7.54 | 5.86 | 29.08 |
|  | Anterior | 15 | 19.30 | 7.15 | 7.95 | 31.11 |
|  | Ipsilateral | 15 | 12.55 | 7.73 | 4.72 | 32.32 |
| Stable | Contralateral | 15 | 11.77 | 4.56 | 6.02 | 21.48 |
|  | Contralateral Diagonal | 15 | 11.14 | 4.86 | 6.29 | 22.98 |
|  | Anterior | 15 | 16.48 | 7.61 | 8.49 | 31.91 |
|  | Ipsilateral | 15 | 7.57 | 3.71 | 3.42 | 16.38 |

Trunk lateral flexion

| Seat | Reaching direction | N | Mean angle [°] | SD | Minimal angle [°] | Maximal angle [°] |
| --- | --- | --- | --- | --- | --- | --- |
| Mobile | Contralateral | 15 | 19.64 | 5.93 | 7.80 | 30.27 |
|  | Contralateral Diagonal | 15 | 20.87 | 5.98 | 10.96 | 29.25 |
|  | Anterior | 15 | 6.71 | 3.64 | 3.49 | 16.59 |
|  | Ipsilateral | 15 | 11.85 | 4.64 | 6.86 | 24.22 |
| Stable | Contralateral | 15 | 19.91 | 8.05 | 7.96 | 35.06 |
|  | Contralateral Diagonal | 15 | 20.09 | 9.32 | 7.24 | 34.3 |
|  | Anterior | 15 | 6.55 | 3.95 | 2.85 | 18.96 |
|  | Ipsilateral | 15 | 12.62 | 6.23 | 5.73 | 25.73 |

Trunk rotation

| Seat | Reaching direction | N | Mean angle [°] | SD | Minimal angle [°] | Maximal angle [°] |
| --- | --- | --- | --- | --- | --- | --- |
| Mobile | Contralateral | 15 | 33.89 | 10.23 | 16.52 | 46.21 |
|  | Contralateral Diagonal | 15 | 27.01 | 7.30 | 10.61 | 40.19 |
|  | Anterior | 15 | 9.96 | 3.22 | 2.81 | 17.55 |
|  | Ipsilateral | 15 | 7.40 | 2.79 | 3.04 | 12.90 |
| Stable | Contralateral | 15 | 38.43 | 7.08 | 25.65 | 51.11 |
|  | Contralateral Diagonal | 15 | 29.92 | 8.70 | 12.94 | 45.19 |
|  | Anterior | 15 | 10.06 | 2.80 | 3.76 | 13.63 |
|  | Ipsilateral | 15 | 10.40 | 4.23 | 4.34 | 12.55 |
