# Supplementary material for: Hip and trunk kinematics during reaching on a mobile and stable seat
Source: PLoS One. 2023 Jul 27;18(7):e0289115. doi: 10.1371/journal.pone.0289115 (PMC10374116; doi:10.1371/journal.pone.0289115)
Supplement: S2 File — (DOCX) [file pone.0289115.s006.docx]

# Supplementary Material

Model comparisons: R-Output and results of the 5-fold, 10 times repeated cross validation performed with the R-package cvTools (Version 0.3.2).

**Hip adduction**

Models:

Mod19: $\log\left( y \right)\sim Condition+Exercise+Maxreach+Oblext_{dom}+\left( 1 | Condition:subID \right)+\left( 1 | Exercise:subID \right)+(1|subID)$

Mod18: $\log\left( y \right)\sim Condition+Exercise+Maxreach+Oblext_{ndom}+Oblext_{dom}+\left( 1 | Condition:subID \right)+\left( 1 | Exercise:subID \right)+(1|subID)$

Mod17: $\log\left( y \right)\sim Condition+Exercise+Maxreach+Oblext_{ndom}+Oblext_{dom}+Multifid_{ndom}+\left( 1 | Condition:subID \right)+\left( 1 | Exercise:subID \right)+(1|subID)$

Table 1: Model comparison for hip adduction.

| Model name | Number of parameters | AIC | BIC | Log likelihood | Deviance | Chi-squared | DF | p-value (> Chi-squared) |
| --- | --- | --- | --- | --- | --- | --- | --- | --- |
| Mod19 | 11 | 129.92 | 159.82 | -53.96 | 107.92 |  |  |  |
| Mod18 | 12 | 131.90 | 164.52 | -53.95 | 107.90 | 0.02 | 1 | 0.90 |
| Mod17 | 13 | 133.66 | 169.00 | -53.83 | 107.66 | 0.24 | 1 | 0.62 |

Note. AIC = Akaike information criterion, BIC = Bayesian information criterion, DF = degrees of freedom

Table 2: Type III analysis of variance table with Satterthwaite's method for model 19.

|  | Sum square | Mean square | Numerator DF | Denominator DF | F value | p-value (>F) |
| --- | --- | --- | --- | --- | --- | --- |
| Condition | 0.21 | 0.21 | 1 | 63.82 | 2.10 | 0.15 |
| Exercise | 16.65 | 5.55 | 3 | 52.62 | 56.51 | < 2.2 e-16 |
| Maxreach | 0.07 | 0.07 | 1 | 100.58 | 0.71 | 0.40 |
| Oblext_dom | 0.69 | 0.69 | 1 | 90.22 | 7.00 | 0.01 |

Note. DF = degrees of freedom, maxreach = maximal reaching distance, oblext_dom = dominant side of M. obliquus externus

Table 3: Type III analysis of variance table with Satterthwaite's method for model 18.

|  | Sum square | Mean square | Numerator DF | Denominator DF | F value | p-value (>F) |
| --- | --- | --- | --- | --- | --- | --- |
| Condition | 0.20 | 0.20 | 1 | 63.48 | 1.99 | 0.16 |
| Exercise | 15.66 | 5.22 | 3 | 54.45 | 52.44 | 4.52 e-16 |
| Maxreach | 0.07 | 0.07 | 1 | 99.33 | 0.66 | 0.42 |
| Oblext_ndom | 0.001 | 0.001 | 1 | 97.38 | 0.01 | 0.93 |
| Oblext_dom | 0.65 | 0.65 | 1 | 87.11 | 6.49 | 0.01 |

Note. DF = degrees of freedom, maxreach = maximal reaching distance, oblext_ndom = non-dominant side of M. obliquus externus, oblext_dom = dominant side of M. obliquus externus

Table 4: Type III analysis of variance table with Satterthwaite's method for model 17.

|  | Sum square | Mean square | Numerator DF | Denominator DF | F value | p-value (>F) |
| --- | --- | --- | --- | --- | --- | --- |
| Condition | 0.21 | 0.21 | 1 | 62.86 | 2.06 | 0.16 |
| Exercise | 13.31 | 4.44 | 3 | 58.37 | 44.05 | 5.26 e-15 |
| Maxreach | 0.08 | 0.08 | 1 | 97.91 | 0.77 | 0.38 |
| Oblext_ndom | 0.0001 | 0.0001 | 1 | 96.46 | 0.001 | 0.98 |
| Oblext_dom | 0.65 | 0.651 | 1 | 86.31 | 6.47 | 0.01 |
| Multifid_ndom | 0.02 | 0.022 | 1 | 99.05 | 0.22 | 0.64 |

Note. DF = degrees of freedom, maxreach = maximal reaching distance, oblext_ndom = non-dominant side of M. obliquus externus, oblext_dom = dominant side of M. obliquus externus, Multifid_ndom = non-dominant side of M. multifidus

Estimated prediction error, obtained from cross-validation: coefficient of variation (CV) = 7.86

**Hip flexion**

Models:

Mod19: $\log\left( y \right)\sim Condition+Exercise+Maxreach+Oblext_{dom}+\left( 1 | Condition:subID \right)+\left( 1 | Exercise:subID \right)+(1|subID)$

Mod18: $\log\left( y \right)\sim Condition+Exercise+Maxreach+Oblext_{ndom}+Oblext_{dom}+\left( 1 | Condition:subID \right)+\left( 1 | Exercise:subID \right)+(1|subID)$

Mod17: $\log\left( y \right)\sim Condition+Exercise+Maxreach+Oblext_{ndom}+Oblext_{dom}+Multifid_{ndom}+\left( 1 | Condition:subID \right)+\left( 1 | Exercise:subID \right)+(1|subID)$

Table 5: Model comparison for hip flexion.

| Model name | Number of parameters | AIC | BIC | Log likelihood | Deviance | Chi-squared | DF | p-value (>Chi-squared) |
| --- | --- | --- | --- | --- | --- | --- | --- | --- |
| Mod19 | 11 | 127.39 | 157.29 | -52.70 | 105.39 |  |  |  |
| Mod18 | 12 | 128.26 | 160.88 | -52.13 | 104.26 | 1.13 | 1 | 0.29 |
| Mod17 | 13 | 128.74 | 164.08 | -51.37 | 102.74 | 1.52 | 1 | 0.22 |

Note. AIC = Akaike information criterion, BIC = Bayesian information criterion, DF = degrees of freedom

Table 6: Type III analysis of variance table with Satterthwaite's method for model 19.

|  | Sum square | Mean square | Numerator DF | Denominator DF | F value | p-value (>F) |
| --- | --- | --- | --- | --- | --- | --- |
| Condition | 3.26 | 3.26 | 1 | 64.84 | 25.25 | 4.21 e-06 |
| Exercise | 7.76 | 2.59 | 3 | 51.81 | 20.00 | 9.61 e-09 |
| Maxreach | 2.15 | 2.15 | 1 | 87.01 | 16.65 | 9.92 e-05 |
| Oblext_dom | 0.001 | 0.001 | 1 | 77.60 | 0.01 | 0.92 |

Note. DF = degrees of freedom, maxreach = maximal reaching distance, oblext_dom = dominant side of M. obliquus externus

Table 7: Type III analysis of variance table with Satterthwaite's method for model 18.

|  | Sum square | Mean square | Numerator DF | Denominator DF | F value | p-value (>F) |
| --- | --- | --- | --- | --- | --- | --- |
| Condition | 2.97 | 2.97 | 1 | 65.75 | 22.71 | 1.08 e-05 |
| Exercise | 7.90 | 2.63 | 3 | 53.76 | 20.17 | 6.92 e-09 |
| Maxreach | 1.78 | 1.78 | 1 | 92.66 | 13.64 | 3.74 e-4 |
| Oblext_ndom | 0.13 | 0.13 | 1 | 101.71 | 1.02 | 0.32 |
| Oblext_dom | 0.02 | 0.02 | 1 | 78.60 | 0.18 | 0.67 |

Note. DF = degrees of freedom, maxreach = maximal reaching distance, oblext_ndom = non-dominant side of M. obliquus externus, oblext_dom = dominant side of M. obliquus externus

Table 8: Type III analysis of variance table with Satterthwaite's method for model 17

|  | Sum square | Mean square | Numerator DF | Denominator DF | F value | p-value (>F) |
| --- | --- | --- | --- | --- | --- | --- |
| Condition | 2.87 | 2.87 | 1 | 64.86 | 22.50 | 1.19 e-05 |
| Exercise | 5.94 | 1.98 | 3 | 56.32 | 15.52 | 1.78 e-07 |
| Maxreach | 1.56 | 1.56 | 1 | 88.36 | 12.22 | 7.40 e-04 |
| Oblext_ndom | 0.19 | 0.19 | 1 | 101.20 | 1.51 | 0.22 |
| Oblext_dom | 0.02 | 0.02 | 1 | 77.76 | 0.16 | 0.69 |
| Multifid_ndom | 0.20 | 0.20 | 1 | 94.60 | 1.65 | 0.21 |

Note. DF = degrees of freedom, maxreach = maximal reaching distance, oblext_ndom = non-dominant side of M. obliquus externus, oblext_dom = dominant side of M. obliquus externus, Multifid_ndom = non-dominant side of M. multifidus

Estimated prediction error, obtained from cross-validation: CV = 16.87

**Hip external rotation**

Models:

Mod19: $\log\left( y \right)\sim Condition+Exercise+Maxreach+Oblext_{dom}+\left( 1 | Condition:subID \right)+\left( 1 | Exercise:subID \right)+(1|subID)$

Mod18: $\log\left( y \right)\sim Condition+Exercise+Maxreach+Oblext_{ndom}+Oblext_{dom}+\left( 1 | Condition:subID \right)+\left( 1 | Exercise:subID \right)+(1|subID)$

Mod17: $\log\left( y \right)\sim Condition+Exercise+Maxreach+Oblext_{ndom}+Oblext_{dom}+Multifid_{ndom}+\left( 1 | Condition:subID \right)+\left( 1 | Exercise:subID \right)+(1|subID)$

Table 9: Model comparison for hip external rotation.

| Model name | Number of parameters | AIC | BIC | Log likelihood | Deviance | Chi-squared | DF | p-value (>Chi-squared) |
| --- | --- | --- | --- | --- | --- | --- | --- | --- |
| Mod19 | 11 | 68.54 | 98.45 | -23.27 | 46.54 |  |  |  |
| Mod18 | 12 | 70.54 | 103.17 | -23.27 | 46.54 | 0.0001 | 1 | 0.99 |
| Mod17 | 13 | 71.75 | 107.09 | -22.89 | 45.75 | 0.79 | 1 | 0.37 |

Note. AIC = Akaike information criterion, BIC = Bayesian information criterion, DF = degrees of freedom

Table 10: Type III analysis of variance table with Satterthwaite's method for model 19.

|  | Sum square | Mean square | Numerator DF | Denominator DF | F value | p-value (>F) |
| --- | --- | --- | --- | --- | --- | --- |
| Condition | 0.21 | 0.21 | 1 | 16.93 | 2.11 | 0.10 |
| Exercise | 1.32 | 0.44 | 3 | 50.94 | 6.56 | 0.001 |
| Maxreach | 0.44 | 0.44 | 1 | 102.72 | 6.58 | 0.01 |
| Oblext_dom | 0.23 | 0.23 | 1 | 81.81 | 3.46 | 0.07 |

Note. DF = degrees of freedom, maxreach = maximal reaching distance, oblext_dom = dominant side of M. obliquus externus

Table 11: Type III analysis of variance table with Satterthwaite's method for model 18.

|  | Sum square | Mean square | Numerator DF | Denominator DF | F value | p-value (>F) |
| --- | --- | --- | --- | --- | --- | --- |
| Condition | 0.20 | 0.20 | 1 | 17.50 | 3.01 | 0.10 |
| Exercise | 1.32 | 0.44 | 3 | 52.88 | 6.50 | 7.95 e-4 |
| Maxreach | 0.43 | 0.42 | 1 | 103.35 | 6.27 | 0.01 |
| Oblext_ndom | 0.0002 | 0.0002 | 1 | 94.07 | 0.003 | 0.96 |
| Oblext_dom | 0.22 | 0.22 | 1 | 79.44 | 3.18 | 0.08 |

Note. DF = degrees of freedom, maxreach = maximal reaching distance, oblext_ndom = non-dominant side of M. obliquus externus, oblext_dom = dominant side of M. obliquus externus

Table 12: Type III analysis of variance table with Satterthwaite's method for model 17

|  | Sum square | Mean square | Numerator DF | Denominator DF | F value | p-value (>F) |
| --- | --- | --- | --- | --- | --- | --- |
| Condition | 0.19 | 0.19 | 1 | 17.47 | 2.78 | 0.11 |
| Exercise | 1.37 | 0.46 | 3 | 56.82 | 6.76 | 5.61 e-04 |
| Maxreach | 0.46 | 0.46 | 1 | 102.20 | 6.78 | 0.01 |
| Oblext_ndom | 0.003 | 0.003 | 1 | 93.24 | 0.04 | 0.84 |
| Oblext_dom | 0.21 | 0.21 | 1 | 78.34 | 3.18 | 0.08 |
| Multifid_ndom | 0.05 | 0.05 | 1 | 101.91 | 0.76 | 0.34 |

Note. DF = degrees of freedom, maxreach = maximal reaching distance, oblext_ndom = non-dominant side of M. obliquus externus, oblext_dom = dominant side of M. obliquus externus, Multifid_ndom = non-dominant side of M. multifidus

Estimated prediction error, obtained from cross-validation: CV = 6.37

**Trunk flexion extension**

Models:

Mod20: $\log\left( y \right)\sim Condition+Exercise+Maxreach+{Erecspin}_{ndom}+Oblext_{ndom}+ \left( 1 | Condition:subID \right)+\left( 1 | Exercise:subID \right)+(1|subID)$

Mod19: $\log\left( y \right)\sim Condition+Exercise+Maxreach+Erecspin_{ndom}+Oblext_{ndom}+Oblext_{dom}+\left( 1 | Condition:subID \right)+\left( 1 | Exercise:subID \right)+(1|subID)$

Mod18: $\log\left( y \right)\sim Condition+Exercise+Maxreach+Erecspin_{ndom}+Erecspin_{dom}+Oblext_{ndom}+Oblext_{dom}+Multifid_{ndom}+\left( 1 | Condition:subID \right)+\left( 1 | Exercise:subID \right)+(1|subID)$

Table 13: Model comparison for trunk flexion extension.

| Model name | Number of parameters | AIC | BIC | Log likelihood | Deviance | Chi-squared | DF | p-value (>Chi-squared) |
| --- | --- | --- | --- | --- | --- | --- | --- | --- |
| Mod20 | 12 | 106.75 | 139.38 | -41.38 | 82.75 |  |  |  |
| Mod19 | 13 | 105.83 | 141.17 | -39.92 | 79.83 | 2.92 | 1 | 0.09 |
| Mod18 | 14 | 107.30 | 145.36 | -39.65 | 79.30 | 0.53 | 1 | 0.47 |

Note. AIC = Akaike information criterion, BIC = Bayesian information criterion, DF = degrees of freedom

Table 14: Type III analysis of variance table with Satterthwaite's method for model 20.

|  | Sum square | Mean square | Numerator DF | Denominator DF | F value | p-value (>F) |
| --- | --- | --- | --- | --- | --- | --- |
| Condition | 0.85 | 0.85 | 1 | 16.55 | 12.96 | 0.002 |
| Exercise | 0.39 | 0.13 | 3 | 53.50 | 1.97 | 0.13 |
| Maxreach | 0.68 | 0.68 | 1 | 94.91 | 10.34 | 0.002 |
| Erecspin_ndom | 0.0002 | 0.0002 | 1 | 99.25 | 0.004 | 0.95 |
| Oblext_ndom | 0.09 | 0.09 | 1 | 82.48 | 1.33 | 0.25 |

Note. DF = degrees of freedom, maxreach = maximal reaching distance, erecspin_ndom = non-dominant side of M. Erector spinae, oblext_ndom = non-dominant side of M. obliquus externus

Table 15: Type III analysis of variance table with Satterthwaite's method for model 19.

|  | Sum square | Mean square | Numerator DF | Denominator DF | F value | p-value (>F) |
| --- | --- | --- | --- | --- | --- | --- |
| Condition | 0.68 | 0.68 | 1 | 16.18 | 11.33 | 0.004 |
| Exercise | 0.33 | 0.11 | 3 | 53.97 | 1.87 | 0.15 |
| Maxreach | 0.56 | 0.56 | 1 | 89.77 | 9.33 | 0.003 |
| Erecspin_ndom | 0.001 | 0.001 | 1 | 94.96 | 0.01 | 0.91 |
| Oblext_dom | 0.19 | 0.19 | 1 | 85.33 | 3.17 | 0.08 |
| Oblext_ndom | 0.14 | 0.14 | 1 | 74.44 | 2.42 | 0.12 |

Note. DF = degrees of freedom, maxreach = maximal reaching distance, erecspin_ndom = non-dominant side of M. erector spinae, oblext_ndom = non-dominant side of M. obliquus externus, oblext_dom = dominant side of M. obliquus externus

Table 16: Type III analysis of variance table with Satterthwaite's method for model 18

|  | Sum square | Mean square | Numerator DF | Denominator DF | F value | p-value (>F) |
| --- | --- | --- | --- | --- | --- | --- |
| Condition | 0.65 | 0.65 | 1 | 16.72 | 10.56 | 0.005 |
| Exercise | 0.36 | 0.12 | 3 | 53.54 | 1.95 | 0.13 |
| Maxreach | 0.43 | 0.43 | 1 | 87.46 | 7.002 | 0.01 |
| Erecspin_ndom | 0.0001 | 0.0001 | 1 | 94.23 | 0.002 | 0.96 |
| Erecspin_dom | 0.03 | 0.03 | 1 | 80.16 | 0.46 | 0.50 |
| Oblext_dom | 0.17 | 0.17 | 1 | 83.05 | 2.82 | 0.10 |
| Oblext_ndom | 0.15 | 0.15 | 1 | 74.23 | 2.39 | 0.13 |

Note. DF = degrees of freedom, maxreach = maximal reaching distance, erecspin_ndom = non-dominant side of M. erector spinae, erecspin_dom = dominant side of M. erector spinae, oblext_ndom = non-dominant side of M. obliquus externus, oblext_dom = dominant side of M. obliquus externus

Estimated prediction error, obtained from cross-validation: CV = 10.36

**Trunk lateral flexion**

Models:

Mod20: $\log\left( y \right)\sim Condition+Exercise+Maxreach+Erecspin_{ndom}+Oblext_{ndom}+\left( 1 | Condition:subID \right)+\left( 1 | Exercise:subID \right)+(1|subID)$

Mod19: $\log\left( y \right)\sim Condition+Exercise+Maxreach+Erecspin_{dom}+Erecspin_{ndom}+Oblext_{ndom}+\left( 1 | Condition:subID \right)+\left( 1 | Exercise:subID \right)+(1|subID)$

Mod18: $\log\left( y \right)\sim Condition+Exercise+Maxreach+Erecspin_{dom}+Erecspin_{ndom}+Oblext_{ndom}+Oblext_{dom}+\left( 1 | Condition:subID \right)+\left( 1 | Exercise:subID \right)+(1|subID)$

Table 17: Model comparison for trunk lateral flexion.

| Model name | Number of parameters | AIC | BIC | Log likelihood | Deviance | Chi-squared | DF | p-value (>Chi-squared) |
| --- | --- | --- | --- | --- | --- | --- | --- | --- |
| Mod20 | 12 | 108.02 | 140.64 | -42.01 | 84.02 |  |  |  |
| Mod19 | 13 | 105.42 | 140.76 | -39.71 | 79.42 | 4.60 | 1 | 0.03 |
| Mod18 | 14 | 107.11 | 145.16 | -39.55 | 79.11 | 0.31 | 1 | 0.57 |

Note. AIC = Akaike information criterion, BIC = Bayesian information criterion, DF = degrees of freedom

Table 18: Type III analysis of variance table with Satterthwaite's method for model 20.

|  | Sum square | Mean square | Numerator DF | Denominator DF | F value | p-value (>F) |
| --- | --- | --- | --- | --- | --- | --- |
| Condition | 0.04 | 0.04 | 1 | 16.31 | 0.59 | 0.46 |
| Exercise | 7.56 | 2.52 | 3 | 52.64 | 40.85 | 8.91 e-14 |
| Maxreach | 0.21 | 0.21 | 1 | 86.32 | 3.43 | 0.07 |
| Erecspin_ndom | 0.01 | 0.01 | 1 | 87.11 | 0.17 | 0.68 |
| Oblext_ndom | 0.02 | 0.02 | 1 | 77.73 | 0.34 | 0.56 |

Note. DF = degrees of freedom, maxreach = maximal reaching distance, erecspin_ndom = non-dominant side of M. Erector spinae, oblext_ndom = non-dominant side of M. obliquus externus

Table 19: Type III analysis of variance table with Satterthwaite's method for model 19.

|  | Sum square | Mean square | Numerator DF | Denominator DF | F value | p-value (>F) |
| --- | --- | --- | --- | --- | --- | --- |
| Condition | 0.01 | 0.01 | 1 | 16.50 | 0.20 | 0.66 |
| Exercise | 6.94 | 2.31 | 3 | 52.70 | 39.71 | 1.49 e-13 |
| Maxreach | 0.06 | 0.06 | 1 | 81.83 | 1.030 | 0.31 |
| Erecspin_dom | 0.26 | 0.26 | 1 | 73.91 | 4.45 | 0.04 |
| Erecspin_ndom | 0.01 | 0.01 | 1 | 82.20 | 0.08 | 0.78 |
| Oblext_ndom | 0.03 | 0.03 | 1 | 75.14 | 0.43 | 0.51 |

Note. DF = degrees of freedom, maxreach = maximal reaching distance, oblext_ndom = non-dominant side of M. obliquus externus, oblext_dom = dominant side of M. erector spinae, erecspin_ndom = non-dominant side of M. erector spinae

Table 20: Type III analysis of variance table with Satterthwaite's method for model 18

|  | Sum square | Mean square | Numerator DF | Denominator DF | F value | p-value (>F) |
| --- | --- | --- | --- | --- | --- | --- |
| Condition | 0.01 | 0.01 | 1 | 16.59 | 0.22 | 0.64 |
| Exercise | 6.70 | 2.23 | 3 | 52.17 | 38.76 | 2.57 e-13 |
| Maxreach | 0.06 | 0.06 | 1 | 80.13 | 1.09 | 0.30 |
| Erecspin_dom | 0.27 | 0.27 | 1 | 73.45 | 4.70 | 0.03 |
| Erecspin_ndom | 0.004 | 0.004 | 1 | 79.41 | 0.06 | 0.80 |
| Oblext_dom | 0.02 | 0.02 | 1 | 90.89 | 0.32 | 0.57 |
| Oblext_ndom | 0.01 | 0.01 | 1 | 72.20 | 0.20 | 0.66 |

Note. DF = degrees of freedom, maxreach = maximal reaching distance, erecspin_ndom = non-dominant side of M. erector spinae, erecspin_dom = dominant side of M. erector spinae, oblext_ndom = non-dominant side of M. obliquus externus, oblext_dom = dominant side of M. obliquus externus

Estimated prediction error, obtained from cross-validation: CV = 12.91

**Trunk rotation**

Models:

Mod20: $\log\left( y \right)\sim Condition+Exercise+Maxreach+Erecspin_{ndom}+Oblext_{ndom}+\left( 1 | Condition:subID \right)+\left( 1 | Exercise:subID \right)+(1|subID)$

Mod19: $\log\left( y \right)\sim Condition+Exercise+Maxreach+Erecspin_{dom}+Erecspin_{ndom}+Oblext_{ndom}+\left( 1 | Condition:subID \right)+\left( 1 | Exercise:subID \right)+(1|subID)$

Mod18: $\log\left( y \right)\sim Condition+Exercise+Maxreach+Erecspin_{dom}+Erecspin_{ndom}+Oblext_{ndom}+Oblext_{dom}+\left( 1 | Condition:subID \right)+\left( 1 | Exercise:subID \right)+(1|subID)$

Table 21: Model comparison for trunk rotation.

| Model name | Number of parameters | AIC | BIC | Log likelihood | Deviance | Chi-squared | DF | p-value (>Chi-squared) |
| --- | --- | --- | --- | --- | --- | --- | --- | --- |
| Mod20 | 12 | 50.21 | 82.83 | -13.10 | 26.21 |  |  |  |
| Mod19 | 13 | 52.19 | 87.53 | -13.10 | 26.19 | 0.02 | 1 | 0.89 |
| Mod18 | 14 | 52.73 | 90.78 | -12.36 | 24.73 | 1.46 | 1 | 0.23 |

Note. AIC = Akaike information criterion, BIC = Bayesian information criterion, DF = degrees of freedom

Table 22: Type III analysis of variance table with Satterthwaite's method for model 20.

|  | Sum square | Mean square | Numerator DF | Denominator DF | F value | p-value (>F) |
| --- | --- | --- | --- | --- | --- | --- |
| Condition | 0.16 | 0.16 | 1 | 18.38 | 4.65 | 0.05 |
| Exercise | 11.43 | 3.81 | 3 | 56.10 | 113.63 | <2.2 e-16 |
| Maxreach | 0.26 | 0.26 | 1 | 81.75 | 7.67 | 0.01 |
| Erecspin_ndom | 0.15 | 0.15 | 1 | 93.81 | 4.43 | 0.04 |
| Oblext_ndom | 0.15 | 0.15 | 1 | 73.45 | 4.41 | 0.04 |

Note. DF = degrees of freedom, maxreach = maximal reaching distance, erecspin_ndom = non-dominant side of M. Erector spinae, oblext_ndom = non-dominant side of M. obliquus externus

Table 23: Type III analysis of variance table with Satterthwaite's method for model 19.

|  | Sum square | Mean square | Numerator DF | Denominator DF | F value | p-value (>F) |
| --- | --- | --- | --- | --- | --- | --- |
| Condition | 0.15 | 0.15 | 1 | 19.18 | 4.33 | 0.05 |
| Exercise | 11.54 | 3.85 | 3 | 56.18 | 112.89 | < 2.2 e-16 |
| Maxreach | 0.24 | 0.24 | 1 | 78.08 | 7.06 | 0.01 |
| Erecspin_dom | 0.001 | 0.001 | 1 | 76.83 | 0.02 | 0.90 |
| Erecspin_ndom | 0.15 | 0.15 | 1 | 92.54 | 4.28 | 0.04 |
| Oblext_ndom | 0.15 | 0.15 | 1 | 72.53 | 4.37 | 0.04 |

Note. DF = degrees of freedom, maxreach = maximal reaching distance, oblext_ndom = non-dominant side of M. obliquus externus, oblext_dom = dominant side of M. erector spinae, erecspin_ndom = non-dominant side of M. erector spinae

Table 24: Type III analysis of variance table with Satterthwaite's method for model 18

|  | Sum square | Mean square | Numerator DF | Denominator DF | F value | p-value (>F) |
| --- | --- | --- | --- | --- | --- | --- |
| Condition | 0.16 | 0.16 | 1 | 19.05 | 4.51 | 0.05 |
| Exercise | 11.85 | 3.95 | 3 | 55.65 | 113.99 | <2 e-16 |
| Maxreach | 0.23 | 0.23 | 1 | 78.45 | 6.63 | 0.01 |
| Erecspin_dom | 0.002 | 0.002 | 1 | 77.96 | 0.07 | 0.79 |
| Erecspin_ndom | 0.12 | 0.12 | 1 | 92.09 | 3.58 | 0.06 |
| Oblext_dom | 0.05 | 0.05 | 1 | 99.01 | 1.38 | 0.24 |
| Oblext_ndom | 0.10 | 0.10 | 1 | 69.78 | 2.83 | 0.10 |

Note. DF = degrees of freedom, maxreach = maximal reaching distance, erecspin_ndom = non-dominant side of M. erector spinae, erecspin_dom = dominant side of M. erector spinae, oblext_ndom = non-dominant side of M. obliquus externus, oblext_dom = dominant side of M. obliquus externus

Estimated prediction error, obtained from cross-validation: CV = 18.66
